# Skeletal Muscle Segmentation at the Level of the Third Lumbar Vertebra (L3) in Low-Dose Computed Tomography: A Lightweight Algorithm

**DOI:** 10.3390/tomography10090111

**Published:** 2024-09-13

**Authors:** Xuzhi Zhao, Yi Du, Haizhen Yue

**Affiliations:** 1 School of Electronic and Information Engineering, Beijing Jiaotong University, Beijing 100044, China; xuzhi_zhao@163.com; 2 Key Laboratory of Carcinogenesis and Translational Research (Ministry of Education/Beijing), Department of Radiation Oncology, Peking University Cancer Hospital & Institute, Beijing 100142, China; haizhenyue@bjcancer.org; 3 Institute of Medical Technology, Peking University Health Science Center, Beijing 100191, China

**Keywords:** skeletal muscles, muscle segmentation, third lumbar vertebra (L3), low-dose CT images, nutritional status

## Abstract

Background: The cross-sectional area of skeletal muscles at the level of the third lumbar vertebra (L3) measured from computed tomography (CT) images is an established imaging biomarker used to assess patients’ nutritional status. With the increasing prevalence of low-dose CT scans in clinical practice, accurate and automated skeletal muscle segmentation at the L3 level in low-dose CT images has become an issue to address. This study proposed a lightweight algorithm for automated segmentation of skeletal muscles at the L3 level in low-dose CT images. Methods: This study included 57 patients with rectal cancer, with both low-dose plain and contrast-enhanced pelvic CT image series acquired using a radiotherapy CT scanner. A training set of 30 randomly selected patients was used to develop a lightweight segmentation algorithm, and the other 27 patients were used as the test set. A radiologist selected the most representative axial CT image at the L3 level for both the image series for all the patients, and three groups of observers manually annotated the skeletal muscles in the 54 CT images of the test set as the gold standard. The performance of the proposed algorithm was evaluated in terms of the Dice similarity coefficient (DSC), precision, recall, 95th percentile of the Hausdorff distance (HD95), and average surface distance (ASD). The running time of the proposed algorithm was recorded. An open source deep learning-based AutoMATICA algorithm was compared with the proposed algorithm. The inter-observer variations were also used as the reference. Results: The DSC, precision, recall, HD95, ASD, and running time were 93.2 ± 1.9% (mean ± standard deviation), 96.7 ± 2.9%, 90.0 ± 2.9%, 4.8 ± 1.3 mm, 0.8 ± 0.2 mm, and 303 ± 43 ms (on CPU) for the proposed algorithm, and 94.1 ± 4.1%, 92.7 ± 5.5%, 95.7 ± 4.0%, 7.4 ± 5.7 mm, 0.9 ± 0.6 mm, and 448 ± 40 ms (on GPU) for AutoMATICA, respectively. The differences between the proposed algorithm and the inter-observer reference were 4.7%, 1.2%, 7.9%, 3.2 mm, and 0.6 mm, respectively, for the averaged DSC, precision, recall, HD95, and ASD. Conclusion: The proposed algorithm can be used to segment skeletal muscles at the L3 level in either the plain or enhanced low-dose CT images.

## 1. Introduction

The cross-sectional area of skeletal muscles at the level of the third lumbar vertebra (L3), as observed in computed tomography (CT) images, is an established imaging biomarker used to assess the nutritional status of patients suffering from sarcopenia or cancer cachexia [1,2,3,4,5,6,7]. Conventionally, the delineation of this area is performed manually by trained radiologists, a process that is time-consuming and prone to errors [8,9,10,11,12]. The need for the accurate and automated segmentation of skeletal muscles at the L3 level in CT images has led to the development of several algorithms [13,14,15,16,17,18,19,20,21,22,23,24,25,26,27,28,29,30,31,32,33,34,35].

These algorithms primarily fall into two categories: deep learning (DL) [13,14,15,16,17,18,19,20,21,22,23,24,25,26,27,28,29] and traditional image-processing schemes [30,31,32,33,34,35]. The DL schemes employ convolutional neural network (CNN) models to facilitate the modelling process. The reported CNN models used for skeletal muscle segmentation include U-Net [13,14,15,16,17,18,19], ResUNet [20], CDFNet [21], FCN [22], FCN-2s-VGG16 [23,24,25], FCN-UNet [26,27], attention V-Net [28], and attention U-Net [29]. However, the training of these CNN models requires a large amount of CT images with manually annotated labels and powerful GPU devices. For example, Nowak et al. [21] used 972 annotated CT images and an NVIDIA Titan RTX GPU for their model training. The substantial time and cost involved in preparing and annotating large datasets, the limitations of hardware resources, and the poor interpretability of DL schemes pose challenges for the clinical application of these CNN models.

On the other hand, the traditional image-processing schemes typically use the shape of the skeletal muscle as prior information to build segmentation pipelines [30,31], registration templates [32,33], reference atlases [34], and random forest features [35]. However, these approaches face challenges due to the irregular nature of skeletal muscle shapes, which can vary significantly depending on the patient’s body size and posture. An evaluation study by Charrière et al. [36] showed that the finite element method proposed in [33], later commercialized as the ABACS module in the SliceOmatic software, underperformed in dealing with L3 CT images featuring irregular muscle shapes. Despite these limitations, the simplicity, lightness, and interpretability of the image-processing schemes make them more straightforward to implement in clinical applications compared to the DL schemes [37].

With the rising concern over the health risks induced by X-ray imaging radiation, low-dose CT scans have become increasingly prevalent in clinical practice [38,39,40]. However, all the abovementioned algorithms [13,14,15,16,17,18,19,20,21,22,23,24,25,26,27,28,29,30,31,32,33,34,35] were designed for CT scans at the standard exposure levels. This leaves a significant gap regarding the segmentation of skeletal muscles at the L3 level in low-dose CT images, which are characterized by compromised image quality [41,42,43].

To this end, this study aims to propose a novel, lightweight algorithm to segment skeletal muscles at the L3 level in low-dose CT images. The proposed algorithm is composed of basic image-processing units and adopts a divide-and-conquer strategy to segment the abdominal and paraspinal muscles separately. The segmentation accuracy of the proposed algorithm is evaluated against the observer-agreed gold standards. It is then directly compared with the existing AutoMATICA algorithm [14], an open source DL-based software. Moreover, the inter-observer variation is investigated to analyze the level of agreement between different observers, and to establish a reference for the performance of algorithmic segmentation.

The highlights of this work are as follows:

(1) A lightweight image-processing algorithm is proposed for the automated segmentation of skeletal muscles at the L3 level in low-dose CT images.
(2) The proposed algorithm is developed using a small, unlabeled dataset and can be efficiently run on a laptop without a graphic processing unit (GPU) device.
(3) The proposed algorithm is validated on both plain (i.e., non-contrast) and contrast-enhanced L3 CT images.
(4) The results indicate that the segmentation accuracy of the proposed algorithm is comparable to that of AutoMATICA, and close to the reference determined with the inter-observer variation.

## 2. Materials and Methods

### 2.1. Patients

A group of 57 patients (38 males and 19 females) were included in this study with the approval of the institutional review board (IRB) at Beijing Cancer Hospital on 2 March 2021 (approval code: 2021KT32). The patients were all diagnosed with rectal cancer and received neoadjuvant chemoradiotherapy at the institution from April 2015 to July 2019. The ages of the patients ranged from 30 to 79, with a median of 62.

### 2.2. Image Acquisition

All the patients underwent pelvic CT scans with a Sensation Open CT scanner (Siemens Healthineers, Erlangen, Germany) for radiotherapy simulation. Each patient was immobilized using customized thermoplastic in the supine posture, and two image series (plain and contrast-enhanced) were acquired. Low-dose image acquisition was performed using the following parameters: a tube voltage of 120 kVp, a mean tube current of 110 mA, a slice thickness of 5 mm, a matrix size of 512 × 512, and a pixel spacing of 1.27 × 1.27 mm^2^. The X-ray exposure level used in this study was lower than the standard reference dose level [44,45] and was comparable to the low-dose protocols [46,47]. In addition, a senior radiologist reviewed all the cases and selected the most representative axial CT image at the L3 level for each of the two image series.

### 2.3. Data Partitioning

To develop the skeletal muscle segmentation algorithm, a total of 30 cases were randomly selected from the whole patient group (30/57, 52.6%) using the Fisher–Yates shuffle [48]. The 60 corresponding CT images at the L3 level from these cases were utilized as a training set to design the algorithm and tune parameters, without the need for manually annotated gold standards. The other 27 cases, which included 54 CT images at the L3 level, served as a test set to evaluate the segmentation accuracy of the proposed algorithm, using manually annotated gold standards for comparison.

### 2.4. Gold Standard

The skeletal muscles in each of the CT images at the L3 level in the test set were manually annotated by three groups of observers, denoted as O1, O2, and O3 hereafter. In each of the groups, a non-medical undergraduate and an oncologist were paired to annotate the skeletal muscles using the ITK-SNAP software (version 3.6.0) [49]. After receiving relevant anatomical training from the expert (phase 1: basic anatomical structure training, 4 h; phase 2: interactive segmentation training, 2 h per undergraduate), the non-medical undergraduates annotated the skeletal muscles in the 54 L3 CT images in a random order. Then, the paired oncologists reviewed and finalized the annotations, making corrections if necessary. This pairing strategy was adopted to optimize time efficiency and ensure accuracy, given the practical limitation that oncologists have limited availability for extensive manual annotations. The manual annotation time was recorded.

**Figure 1:**
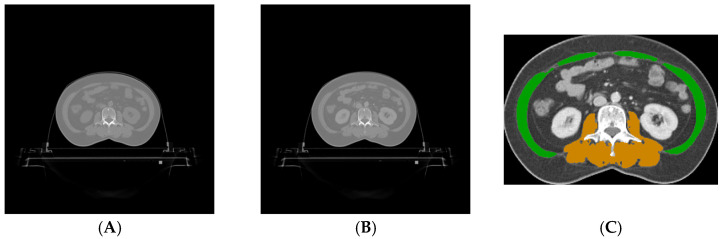
Both the plain (**A**) and contrast-enhanced (**B**) CT images at the third lumbar vertebral level of a case in the test set. The gray scales of the CT images are the same. The corresponding gold standard on the contrast-enhanced CT image (**C**) includes both the abdominal muscles (green) and the paraspinal muscles (orange). For better visualization, the annotated image was cropped, and the gray scale was adjusted.

A consensus gold standard was generated for each CT image at the L3 level in the test set by using a majority voting scheme [50]. This scheme assigned a pixel to the highest class on which at least two groups of observers agreed. Figure 1 shows the CT images at the L3 level of a case in the test set and the gold standard on the contrast-enhanced CT image.

### 2.5. Skeletal Muscle Segmentation

**Figure 2:**
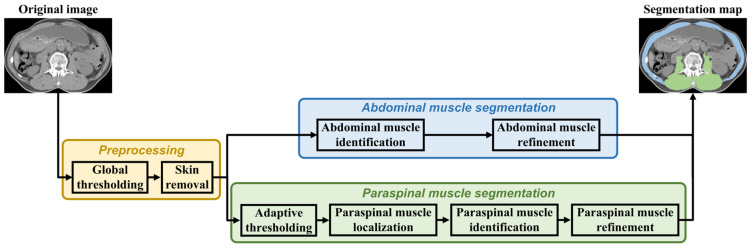
Overall workflow of the proposed skeletal muscle segmentation algorithm.

The proposed skeletal muscle segmentation algorithm was adapted from the algorithm presented in [51]. Figure 2 shows the overall workflow of the proposed algorithm. The workflow consists of three main components: preprocessing, abdominal muscle segmentation, and paraspinal muscle segmentation [52]. Note that the abdominal muscle segmentation and paraspinal muscle segmentation were performed concurrently, which could lead to improved efficiency compared to that when performing them sequentially.

#### 2.5.1. Preprocessing

**Figure 3:**
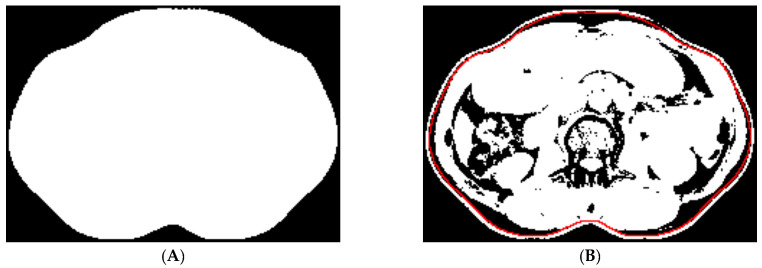
(**A**) The body region on the original image. (**B**) We used the most probable distance (red contour) to remove the skin tissue pixels.

(a) Global thresholding: This step aims to remove most pixels belonging to adipose tissue. The original image was first segmented with a given pair of lower and upper thresholds, −29 and 150 Hounsfield unit (HU), respectively, which were considered the range of standard skeletal muscle CT numbers [53].

(b) Skin removal: This step aims to remove skin tissue pixels for further analysis. Connected component analysis was used to identify the body region from the non-air pixels in the original image (Figure 3A). A Chebyshev distance map was generated from the body region. The most probable distance was determined by locating the isocontour that intersected the highest number of zero-valued pixels on the global thresholding segmented image (Figure 3B). The pixels outside and on the isocontour were then removed. Connected component analysis was then used to filter out the small regions.

#### 2.5.2. Abdominal Muscle Segmentation

**Figure 4:**
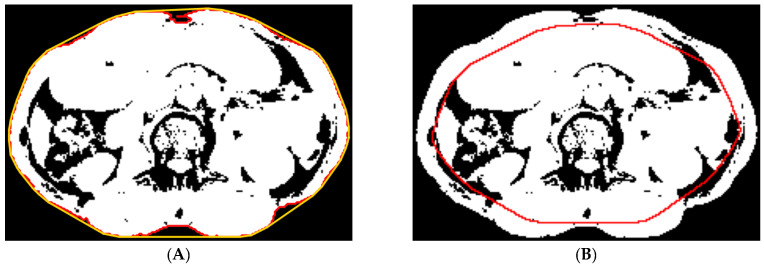
(**A**) The contour (red) and the corresponding convex hull (gold) of the segmented region. (**B**) We used the most probable distance (red contour) to estimate the inside boundary of the abdominal muscles.

**Figure 5:**
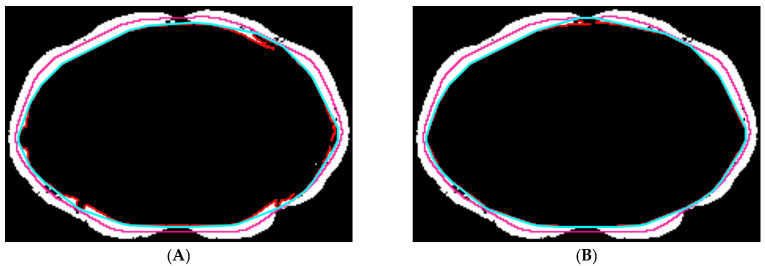
We used the convex hull (cyan) of the largest connected dark region (red) inside the isocontour (pink) to refine the inner profile of the abdominal muscles. The first process and the second process of abdominal muscle refinement are shown in (**A**) and (**B**), respectively.

(a) Abdominal muscle identification: The aim here is to identify the thin layer of abdominal muscles. The convex hull of the contour of the segmented region in the previous step was found (Figure 4A). Then, a Chebyshev distance map was generated from the convex hull. Inside the segmented region, the most probable distance was determined by locating the isocontour that intersected the highest number of zero-valued pixels (Figure 4B). The pixels inside and on the isocontour were removed.

(b) Abdominal muscle refinement: This step refines the segmented abdominal muscles through an iterative process. Using the Chebyshev distance map from the previous step, the isocontour that intersected the highest number of one-valued pixels was located. For the pixels inside the isocontour, the convex hull of the largest connected dark region was extracted. The pixels inside and on the convex hull were then removed (Figure 5). This process was repeated until the number of removed pixels reached zero. Lastly, the region belonging to paraspinal muscles was removed using the result of paraspinal muscle segmentation obtained in the next subsection.

#### 2.5.3. Paraspinal Muscle Segmentation

**Figure 6:**
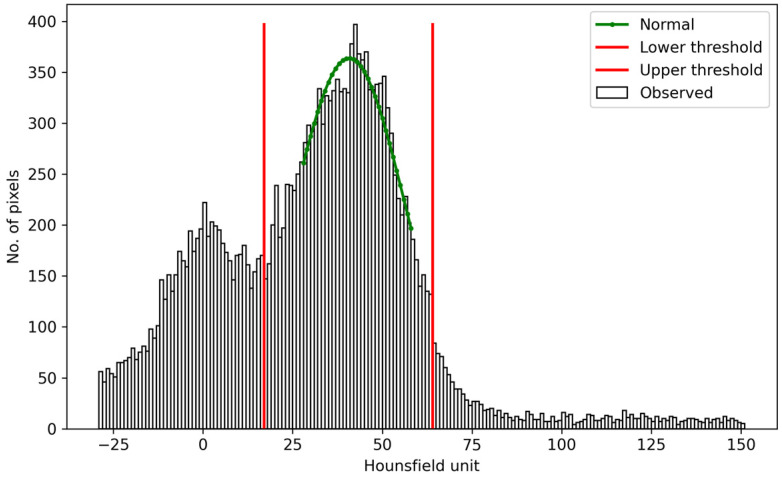
A normal distribution (green) was fitted to the peak region of the histogram to determine the adaptive lower and upper thresholds (red) for double thresholding.

**Figure 7:**
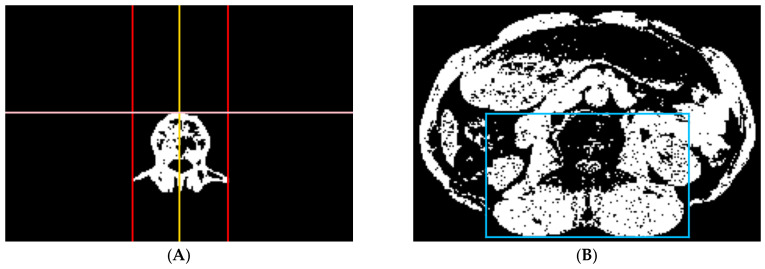
We used the left, right (red), and upper (pink) bounds and the vertical center line (yellow) of the vertebra (**A**) to localize the paraspinal muscle region (blue bounding box) (**B**).

**Figure 8:**
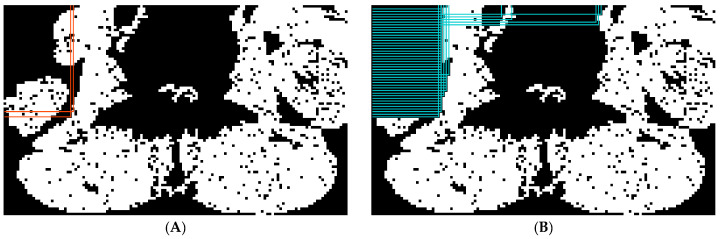
Examples showing the generation of the first form (**A**) and second form (**B**) of adaptive rectangular regions on the upper left corner of the paraspinal muscle localization image. By removing pixels inside the rectangular regions, the paraspinal muscles were identified.

**Figure 9:**
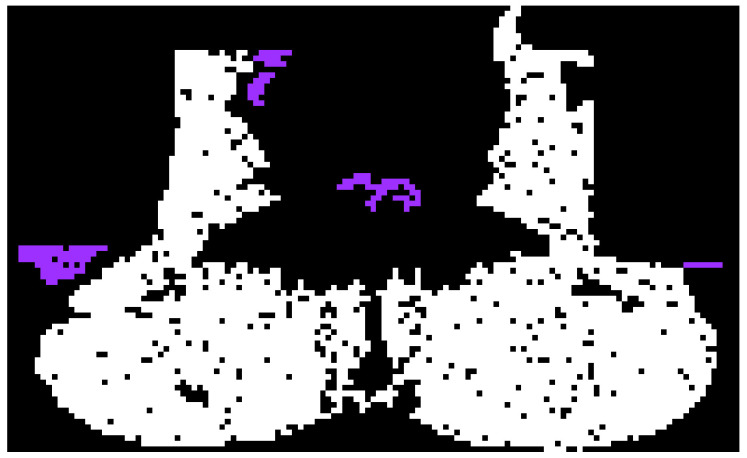
By removing out the non-muscle regions with specific locations and small sizes (purple), the paraspinal muscles were refined.

(a) Adaptive thresholding: This step aims to determine the paraspinal muscle candidates. For the skin-removed body pixels in the preprocessed results, a normal distribution was fitted to the peak of the pixel value histogram to obtain the mean value μ and the standard deviation σ. Adaptive thresholds were then empirically set to be μ – 1.5σ and μ + 1.5σ (confidence coefficient = 86.6%) (Figure 6), which were used as the lower and upper segmentation thresholds. The pixels with values in between were segmented.

(b) Paraspinal muscle localization: This step is designed to find a bounding box enclosing the entire paraspinal muscles for further analysis. The L3 vertebra region was identified by using the connected component analysis from the bone tissue pixels in the original image (Figure 7A). A bounding box was obtained based on the vertebra region using the following methods. The upper border of the bounding box was determined by the upper bound of the vertebra region. The lower border of the bounding box was determined by the bottom-most pixel of the paraspinal muscle candidates. The left and right borders of the bounding box were determined by shifting the vertical center line of the vertebra (yellow line in Figure 7A) to the left and right directions by two times the greater distance of the left and right bounds of the vertebra region from the vertical center line (Figure 7B). Connected component analysis was then used to filter out the small regions in the bounding box.

(c) Paraspinal muscle identification: The aim here is to discern paraspinal muscles from both the muscle and non-muscle tissues. A series of rectangular boxes were generated adaptively in the upper left and upper right corners of the bounding box. Some fixed-length vertical lines were set in the upper left corner of the image along the x-direction, and the distance between the adjacent lines was equal to the physical size of the pixel. If the pixel value of the endpoint of any line was zero, a horizontal line was generated from the left border to the endpoint. The horizontal line then continuously moved downwards until the endpoint belonged to the muscle tissue pixels (Figure 8A). A series of boxes were generated and the pixels inside the boxes were removed. A similar process was implemented but starting with fixed-length horizontal lines along the y-direction, and the corresponding vertical lines were generated and continuously moved to the right (Figure 8B). Two similar processes were implemented in the upper right corner of the image as well.

(d) Paraspinal muscle refinement: The goal of this step is to refine the segmented paraspinal muscles. Connected component analysis was used, and criteria regarding the location and size of the regions were enforced to remove the non-muscle regions (Figure 9). The regions located at the identified vertebra region were removed. The regions near the top, left, and right borders were removed. The small regions were removed. The holes in the image were filled.

Finally, the results of both the abdominal muscle segmentation and the paraspinal muscle segmentation were combined to obtain the complete skeletal muscles. The pseudocode for the proposed algorithm is provided in the Supplementary Materials.

### 2.6. Comparison Study

The proposed algorithm was compared with AutoMATICA [14] for skeletal muscle segmentation in low-dose CT images at the L3 level. AutoMATICA is based on a fully supervised U-Net model, which was trained and validated on a dataset of 804 annotated L3 CT images. These images were selected from 804 regular-dose abdominal CT scans acquired from various patient populations, including critically ill patients, patients with pancreatic cancer, and so on.

Both the proposed algorithm and AutoMATICA were executed on a laptop with an Intel(R) Core(TM) i7-10750H CPU @ 2.60 GHz (manufactured by Intel Corporation, Santa Clara, CA, USA) and an NVIDIA GTX 1650Ti GPU with 4 GB of memory (manufactured by NVIDIA Corporation, Santa Clara, CA, USA). The proposed algorithm was tested solely on the CPU, whereas AutoMATICA was tested on both the CPU and the GPU. The running time of each algorithm on the test set was recorded as an indicator of computation complexity.

### 2.7. Performance Evaluation

For objective evaluation, quantitative metrics including Dice similarity coefficient (DSC), precision, recall, 95th quantile of the Hausdorff distance (HD95), and average surface distance (ASD) were used. The DSC, precision and recall measure the pixel-wise overlap of the segmented and reference regions, while the HD95 and ASD estimate the distance between the segmented and reference boundaries.

The DSC, precision, and recall are defined as

(1) $$\mathrm{DSC}(A,\;B)\;=\;\frac{2\left|A\cap B\right|}{\left|A\right|+\left|B\right|}\times 100\%$$

(2) $$\mathrm{precision}(A,\;B)\;=\;\frac{\left|A\cap B\right|}{\left|A\right|}\times 100\%$$

(3) $$\mathrm{recall}(A,\;B)\;=\;\frac{\left|A\cap B\right|}{\left|B\right|}\times 100\%$$

where *A* is the segmented result and *B* is the corresponding gold standard.

The HD95 and ASD are defined below,

(4) $$\mathrm{HD}95(A,\;B)\;=\;\mathrm{percentile}(\underset{a\in \partial A}{\max}\{\underset{b\in \partial B}{\min}||a-b||\}\cup \underset{b\in \partial B}{\max}\{\underset{a\in \partial A}{\min}||b-a||\},95\mathrm{th})$$

(5) $$\mathrm{ASD}(A,\;B)\;=\;\frac{1}{\left|\partial A|+|\partial B\right|}(\sum_{a\in \partial A}\left\{\underset{b\in \partial B}{\min}||a-b||\right\}+\sum_{b\in \partial B}\left\{\underset{a\in \partial A}{\min}||b-a||\right\})$$

where point *a* is on the surface *∂A* of the segmented result *A*, point *b* is on the surface *∂B* of the gold standard *B*, and ||⋅|| is the Euclidean norm of the points *a* and *b*.

To evaluate the segmentation accuracy of the proposed algorithm and AutoMATICA, two metric sets (DSCs, precisions, recalls, HD95s, and ASDs) were computed. The first and second sets were calculated by comparing the segmentation results obtained by each algorithm with the gold standards for plain CT images and contrast-enhanced CT images in the test set, respectively. The averaged metrics over the different image types were then calculated and denoted as summary results.

To evaluate the inter-observer variation, three metric sets were computed. The first, second, and third sets were calculated by comparing the manual annotations made by O1, O2, and O3 with the gold standards for all the CT images in the test set, respectively. The reference for the performance of algorithmic segmentation was determined by calculating the averaged metrics over the different groups of observers.

### 2.8. Statistical Analysis

The Wilcoxon signed-rank test [54] was used to compare the summary segmentation accuracy on the test set between the proposed algorithm and AutoMATICA. The significance level was set to 0.05.

## 3. Results

### 3.1. Segmentation Accuracy Comparison with AutoMATICA

Table 1 lists the segmentation accuracy of both the proposed algorithm and AutoMATICA in the test set. The results of statistical analysis are also shown. The proposed algorithm outperformed AutoMATICA in terms of precision and HD95 (all *p* < 0.01), but it performed worse than AutoMATICA in terms of DSC and recall (all *p* < 0.01). The proposed algorithm showed a comparable performance to AutoMATICA in terms of ASD (*p* > 0.05). Additionally, for both the proposed algorithm and AutoMATICA, the differences in segmentation accuracy between the plain and contrast-enhanced CT images were within 0.2%, 0.5%, 0.5%, 0.9 mm, and 0 mm, respectively, for the averaged Dice, precision, recall, HD95, and ASD.

**Figure 10:**
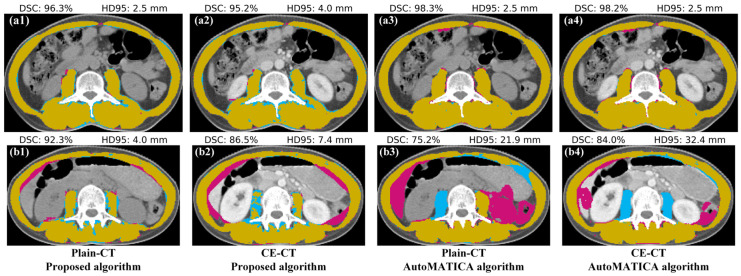
Demonstration of the skeletal muscle segmentation results using the proposed algorithm and the AutoMATICA algorithm. (**a1**–**a4**) Both the algorithms perform well on the plain and contrast-enhanced CT images of a patient case; (**b1**–**a4**) both the algorithms perform poorly on the plain and contrast-enhanced CT images of another patient case. The algorithm segmentation result is shown in pink region, the gold standard is shown in the blue region, and the overlap is shown in the yellow region. Abbreviation: DSC, dice similarity coefficient; HD95, 95th quantile of the Hausdorff distance; Plain-CT, plain CT images; CE-CT, contrast-enhanced CT images.

Figure 10 shows the results of eight skeletal muscle segmentations compared with the corresponding gold standards. The DSC and HD95 are also given for reference. The first four subfigures [Figure 10(a1–a4)] represent a patient case where both the proposed algorithm and AutoMATICA demonstrate a good performance in the plain and contrast-enhanced CT images, with highly overlapped skeletal muscle segmentation results and gold standards. AutoMATICA outperformed the proposed algorithm in terms of segmenting the paraspinal muscles, resulting in better DSC and HD95 values. In contrast, the remaining four subfigures [Figure 10(b1–b4)] represent another patient case where both the algorithms demonstrate a poor performance due to the inaccurate segmentation of the abdominal and paraspinal muscles. Compared to the proposed algorithm, AutoMATICA showed a higher number of pixels classified as incorrect categories, which resulted in lower values for DSC and HD95.

### 3.2. Inter-Observer Variation

**Figure 11:**
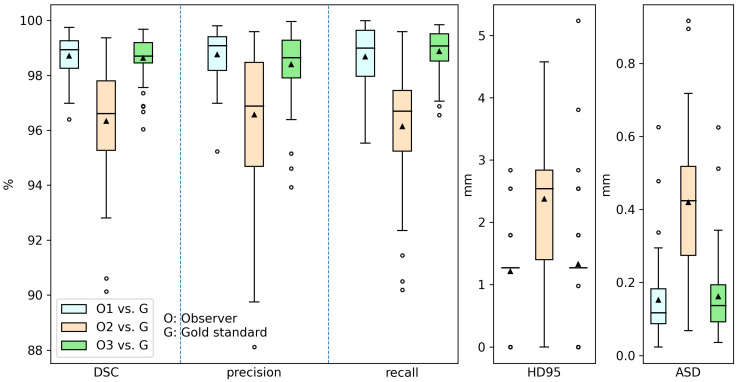
Boxplots of Dice similarity coefficient, precision, recall, 95th percentile of the Hausdorff distance, and average surface distance of inter-observer variation in the test set. The boxes report the first quartile, median, and third quartile; the whiskers extending from the boxes indicate variability outside the first and third quartiles; the outliers are plotted as individual points beyond the whiskers; and the triangles report the mean values.

Figure 11 shows the distributions of inter-observer variation in DSC, precision, recall, HD95, and ASD, respectively. We can see that O1 and O3 showed greater agreement with the gold standards compared to those of O2. The reference metrics determined with the averaged DSC, precision, recall, HD95, and ASD over O1, O2, and O3, were 97.9 ± 1.7%, 97.9 ± 1.9%, 97.9 ± 1.9%, 1.6 ± 1.0 mm, and 0.2 ± 0.2 mm, respectively. The difference between the summary metrics of the proposed algorithm and the reference metrics were 4.7%, 1.2%, 7.9%, 3.2 mm, and 0.6 mm, respectively, for DSC, precision, recall, HD95, and ASD.

### 3.3. Time Cost

Table 2 lists the manual annotation times of O1, O2, and O3, as well as the running times of both the proposed algorithm and AutoMATICA. Compared with manual annotation, the computer algorithm reduced the processing time by at least 140 times. Moreover, the averaged running time of the proposed algorithm on the CPU was approximately one-fifth that of AutoMATICA on the CPU, and approximately two-thirds that of AutoMATICA on the GPU.

## 4. Discussion

The cross-sectional area of skeletal muscles measured from L3 CT images is an established imaging biomarker to assess nutritional status in patients with sarcopenia and cancer [1,2,3,4,5,6,7]. In this study, we proposed a lightweight image-processing algorithm to achieve the automated segmentation of skeletal muscles at the L3 level in low-dose CT images. This algorithm was adapted from our preliminary study [51]. Compared to the previous version, we made several optimizations to enhance its generalization across different body types and skeletal muscle morphologies, using data from a larger patient group with two types of CT images. Additionally, this study provides a more detailed description of the algorithm’s steps. The performance of the proposed algorithm was evaluated on a testing group of 27 patients in comparison with that of the open source DL-based AutoMATICA algorithm.

For algorithm development, the proposed algorithm exhibited advantages over AutoMATICA in two aspects. First, the required data volume was much smaller. Herein, we only used 60 unannotated L3 CT images, while AutoMATICA was trained on 804 annotated L3 CT images. Despite the significant discrepancy in required data size, the results in Table 1 indicated that the segmentation accuracy of the proposed algorithm was comparable to that of AutoMATICA. Second, the computational complexity was significantly reduced. The proposed algorithm was composed of basic image-processing units, while AutoMATICA was based on a complex CNN model. The results in Table 2 showed that the averaged running time of the proposed algorithm on the CPU was over five times shorter than that of AutoMATICA on the CPU, and even shorter than that of AutoMATICA on the GPU for processing one L3 CT image in the test set.

For performance evaluation, three sets of manual annotations of skeletal muscles on the L3 CT images from different observers were collected to evaluate the segmentation accuracy of the proposed algorithm in an unbiased manner, and to establish a reliable estimate of the reference for algorithm performance. By comparing the segmentation results of the proposed algorithm with the consensus gold standards derived from these three sets of manual annotations, we aimed to reduce the potential for biased evaluations, which is a frequent issue when relying on a single-observer-annotated ground truth [13,14,15,16,17,18,19,20,21,22,23,24,25,26,27,28,29,30,31,32,33,34,35]. Moreover, the inter-observer variation was evaluated to provide a reference for algorithm performance, i.e., the human-level upper limit of the segmentation tasks. The results in Section 3.2 indicated that the segmentation accuracy of the proposed algorithm was close to the reference determined with the inter-observer variation.

Compared with the previous studies [13,14,15,16,17,18,19,20,21,22,23,24,25,26,27,28,29,30,31,32,33,34,35], one of the highlights of this study was that we validated the proposed algorithm on both plain and contrast-enhanced L3 CT images. As shown in Figure 10, the injection of contrast agent induced substantial pixel value shifts in not only the blood vessels, but also the abdominal organs, including the intestines, kidneys, and liver. This pixel value shift posed a serious challenge for skeletal muscle segmentation algorithm. Nevertheless, the results in Table 1 showed that the proposed algorithm achieved satisfactory segmentation accuracy when processing the L3 CT images acquired with plain and contrast-enhanced scanning protocols, with the DSCs all exceeding 86%.

The proposed algorithm’s lightweight and interoperable design offers distinct benefits for clinical applications. Unlike state-of-the-art (SOTA) deep learning models [17,18,19,20,21,28,29], which typically require substantial computational resources and are often constrained to running on GPUs, the proposed algorithm is designed to run efficiently on a standard laptop without the need for GPU devices. This makes it particularly suitable for clinical settings where hardware resources may be limited. Furthermore, the SOTA deep learning models often suffer from poor interpretability, making it difficult to visualize and understand the specific processes involved in image segmentation. In contrast, the proposed algorithm is fully interpretable, providing viewable results at each step. This transparent aligns well with the demand for interoperable and reliable algorithms in clinical practice. In terms of segmentation accuracy, the SOTA deep learning models [17,18,19,20,21,28,29] reported averaged DSC values of 0.93, 0.9379, 0.94, 0.92, 0.95, 0.9577, and 0.939, respectively. The proposed algorithm achieved an averaged DSC of 0.932, demonstrating that its segmentation accuracy is comparable to these advanced models.

The limitation of this study comes in two aspects. First, limited by the IRB scope, we only collected low-dose CT images from 57 patients. The data volume used for algorithm development and evaluation was relatively small. Second, the proposed algorithm was developed for segmenting skeletal muscles in a representative axial CT image at the L3 level for each image series, although it can be extended to three-dimensional images with necessary adaptation. Future studies are needed to address these issues.

## 5. Conclusions

The proposed lightweight image-processing algorithm can be used to segment skeletal muscles at the L3 level in either the plain or enhanced low-dose CT images. Further studies are warranted to demonstrate that the proposed algorithm can serve as a computer-aided tool for assessing the nutritional status in patients with rectal cancer or other malignancies.

## Figures and Tables

**Table 1:** Comparison of the segmentation accuracy of both the proposed algorithm and the AutoMATICA algorithm in the test set. All the values are reported as MEAN ± SD.

Metrics ^†^	Algorithm	Image Series		Summary	*p*-Value *
		Plain-CT	CE-CT		
DSC (%)	Proposed	93.2 ± 1.6	93.2 ± 2.2	93.2 ± 1.9	<0.01
	AutoMATICA	94.0 ± 4.6	94.2 ± 3.4	94.1 ± 4.1	
precision (%)	Proposed	97.0 ± 2.2	96.5 ± 3.5	96.7 ± 2.9	<<0.01
	AutoMATICA	92.5 ± 6.1	93.0 ± 4.8	92.7 ± 5.5	
recall (%)	Proposed	89.7 ± 3.0	90.2 ± 2.9	90.0 ± 2.9	<<0.01
	AutoMATICA	95.7 ± 4.0	95.7 ± 4.0	95.7 ± 4.0	
HD95 (mm)	Proposed	4.6 ± 1.1	4.9 ± 1.5	4.8 ± 1.3	<0.01
	AutoMATICA	6.9 ± 4.8	7.8 ± 6.5	7.4 ± 5.7	
ASD (mm)	Proposed	0.8 ± 0.2	0.8 ± 0.3	0.8 ± 0.2	>0.05
	AutoMATICA	0.9 ± 0.6	0.9 ± 0.6	0.9 ± 0.6	

* Statistical analyses were conducted to compare the summary results of the proposed algorithm with those of the *AutoMATICA* algorithm. ^†^ Abbreviation: DSC, dice similarity coefficient; HD95, 95th quantile of the Hausdorff distance; ASD, average surface distance; Plain-CT, plain CT images; CE-CT, contrast-enhanced CT images.

**Table 2:** Comparison of the time cost for the manual annotation of skeletal muscles by three groups of observers and skeletal muscle segmentation using both the proposed algorithm and the AutoMATICA algorithm. The manual annotation time costs are reported as MEAN, while the algorithm-based time costs are reported as MEAN ± SD. Note that the time cost is measured in milliseconds.

Items ^†^	O1	O2	O3	Proposed (CPU) *	AutoMATICA (CPU) *	AutoMATICA (GPU) *
Plain-CT	-	-	-	289 ± 37	1416 ± 43	447 ± 39
CE-CT	-	-	-	316 ± 45	1681 ± 40	448 ± 42
Summary	334,444	212,222	455,556	303 ± 43	1548 ± 140	448 ± 40

^†^ Abbreviation: Plain-CT, plain CT images; CE-CT, contrast-enhanced CT images; O, observer. * (CPU) indicates the algorithm’s running time on the CPU, and (GPU) indicates the algorithm’s running time on the GPU.

## Data Availability

The datasets generated and/or analyzed during the current study are not publicly available due to patient privacy but are available from the corresponding author on reasonable request.

## Supplementary Materials

The following supporting information can be downloaded at: https://www.mdpi.com/article/10.3390/tomography10090111/s1, Table S1: Pseudocode of the proposed lightweight image-processing algorithm.
