# Supplementary material for: Skeletal Muscle Segmentation at the Level of the Third Lumbar Vertebra (L3) in Low-Dose Computed Tomography: A Lightweight Algorithm
Source: Tomography. 2024 Sep 13;10(9):1513–26. doi: 10.3390/tomography10090111 (PMC11435900; doi:10.3390/tomography10090111)
Supplement: Supplementary file 1 [file tomography-10-00111-s001.zip › tomography-3165811-supplementary.pdf]

Pseudocode of the proposed lightweight image-processing algorithm

Algorithm 1: Proposed Lightweight Skeletal Muscle Segmentation Algorithm

Input: Low-dose CT image at the L3 level

1. Preprocessing

1.1 Global thresholding:

Segment the input image using global thresholds [-29, 150] HU.

1.2 Skin removal:

1.2.1 Apply connected component analysis to identify the body region in the input image.

1.2.2 Generate a Chebyshev distance map from the body region.

1.2.3 Locate the isocontour that intersects the highest number of zero-valued pixels on the segmented result from 1.1.

1.2.4 Remove pixels outside and on the isocontour.

1.2.5 Apply connected component analysis to filter out small regions.

2. Abdominal Muscle Segmentation

2.1 Abdominal muscle identification:

2.1.1 Extract the convex hull of the contour of the segmented result from 1.2.

2.1.2 Generate a Chebyshev distance map from the convex hull.

2.1.3 Locate the isocontour that intersects the highest number of zero-valued pixels on the segmented result from 1.2.

2.1.4 Remove pixels inside and on the isocontour.

2.2 Abdominal muscle refinement:

2.2.1 Use the Chebyshev distance map from 2.1.2 to locate the isocontour that intersects the highest number of one-valued pixels on the segmented result from 2.1.

2.2.2 Extract the convex hull of the largest connected dark region inside the isocontour.

2.2.3 Remove pixels inside and on the convex hull.

2.2.4 Repeat steps 2.2.2-2.2.3 until the number of removed pixels reached zero.

3. Paraspinal Muscle Segmentation

3.1 Adaptive thresholding:

3.1.1 Fit a normal distribution to the peak of the pixel value histogram from the pixels corresponding to the segmented result from 1.2 to obtain the mean (μ) and standard deviation (σ).

3.1.2 Set adaptive thresholds to [μ - 1.5σ, μ + 1.5σ] and segment pixels within this range.

3.2 Paraspinal muscle localization:

3.2.1 Identify the L3 vertebra region using connected component analysis on bone tissue pixels in the input image.

3.2.2 Define a bounding box around the paraspinal muscles based on the identified L3 vertebra region:

- Upper border: Upper bound of vertebra region.
- Lower border: Bottommost pixel of the segmented result from 3.1.
- Left/Right borders: Shift the vertical centerline of the vertebra region by twice the greater distance between the vertebra region's left/right bounds and the centerline.

3.2.3 Apply connected component analysis to filter out small regions within the bounding box.

3.3 Paraspinal muscle identification:

3.3.1 Generate adaptive rectangular boxes in the upper left corner of the bounding box obtained in 3.2:

3.3.1.1 Generate fixed-length vertical lines along the *x*-direction.

3.3.1.2 If the endpoint of a vertical line is zero, generate a horizontal line from the left border to the endpoint and move it downwards until the endpoint of the horizontal line intersects muscle tissue.

3.3.1.3 Generate fixed-length horizontal lines along the *y*-direction.

3.3.1.4 If the endpoint of a horizontal line is zero, generate a vertical line from the upper border to the endpoint and move it to the right until the endpoint of the vertical line intersects muscle tissue.

3.3.2 Use steps similar to 3.3.1.1-3.3.1.4 to generate adaptive rectangular boxes in the upper right corner of the bounding box.

3.3.3 Remove all pixels inside the generated rectangular boxes.

3.4 Paraspinal muscle refinement:

3.4.1 Apply connected component analysis to remove non-muscle regions in the segmented result from 3.3:

- Remove regions located at the vertebra region identified in 3.2.1.
- Remove regions near the top, left and right borders of the bounding box.

3.4.2 Fill any holes within the remaining muscle regions.

4. Combine the segmented abdominal and paraspinal muscles to obtain the complete skeletal muscles.

End Algorithm

Output: Segmented skeletal muscles
